# A Structural Equation Modeling Approach to Examining Factors Influencing Outcomes with Cochlear Implant in Mandarin-Speaking Children

**DOI:** 10.1371/journal.pone.0136576

**Published:** 2015-09-08

**Authors:** Yuan Chen, Lena L. N. Wong, Shufeng Zhu, Xin Xi

**Affiliations:** 1 Clinical Hearing Sciences (CHearS) Laboratory, Division of Speech and Hearing Sciences, The University of Hong Kong, Hong Kong, China; 2 Department of Electrical and Electronic Engineering, South University of Science and Technology of China, Shenzhen, China; 3 Department of Otolaryngology Head and Neck Surgery, Chinese PLA General Hospital, Beijing, China; University of South Florida, UNITED STATES

## Abstract

**Objective:**

To examine the direct and indirect effects of demographical factors on speech perception and vocabulary outcomes of Mandarin-speaking children with cochlear implants (CIs).

**Methods:**

115 participants implanted before the age of 5 and who had used CI before 1 to 3 years were evaluated using a battery of speech perception and vocabulary tests. Structural equation modeling was used to test the hypotheses proposed.

**Results:**

Early implantation significantly contributed to speech perception outcomes while having undergone a hearing aid trial (HAT) before implantation, maternal educational level (MEL), and having undergone universal newborn hearing screening (UNHS) before implantation had indirect effects on speech perception outcomes via their effects on age at implantation. In addition, both age at implantation and MEL had direct and indirect effects on vocabulary skills, while UNHS and HAT had indirect effects on vocabulary outcomes via their effects on age at implantation.

**Conclusion:**

A number of factors had indirect and direct effects on speech perception and vocabulary outcomes in Mandarin-speaking children with CIs and these factors were not necessarily identical to those reported among their English-speaking counterparts.

## Introduction

A number of factors have been found to account for the diversity of speech perception and vocabulary skills after cochlear implantation. These variables include age at implantation [1, 2], preoperative residual hearing [3], duration of Cochlear implant (CI) use [4], device characteristics such as speech coding strategies and dynamic range [5], cognitive ability [6], mode of communication [7], and parental education [6].

The identification of these predictive factors are of importance for determination of CI candidacy and habilitation. However, most studies were conducted in English-speaking children; it is not uncertain whether the predictive value of these factors is the same in Mandarin-peaking children considering the linguistic, cultural and social differences between the Mandarin-speaking and English-speaking communities. First, Mandarin is a tonal language, in which tone is lexically meaningful. Accurate perception of tone information requires the representation of temporal fine structure information (i.e., short-term changes in amplitude over short periods of time) [8]. However, this information is usually largely discarded in most current CI systems. Second, in Mainland China, due to under-spending on national health care and a large population, there is a lack of hearing health care professionals (fewer than one audiologist per million people), the lack of an effective universal newborn hearing screening (UNHS) system and a systematic follow-up process [9]. As a result, intervention is usually not provided until Mandarin-speaking children are about 2 to 3 years of age, and many children do not undergo a hearing aid trial (HAT) before implantation [10, 11]. In addition, unlike many parents of English-speaking children with CIs, who are high school or college graduates [12], the majority of parents of children with CIs in mainland China tend to have lower educational levels (i.e., lower than high-school level) [11]. Previous studies have shown that late age at implantation, failure to undergo a HAT before implantation, and low maternal education level (MEL) are significantly related to poor speech perception in Mandarin-speaking children with CIs [11].

In contrast, some linguistic and cultural factors may contribute positively to speech perception and vocabulary development in Mandarin-speaking children. First, compared to English, vowels in Chinese make a much greater contribution to sentence intelligibility [13], and children seem to perceive vowels better and acquire them earlier than consonants [14]. Thus, early speech perception and vocabulary development in Mandarin-speaking children may follow a different course from that of English-speaking children. Second, the stigma associated with deafness as well as the fragmentation of services for individuals with HI in China enhances the desire to “live life in the mainstream” [9]. As a result, the majority of children with CIs in Mainland China use the oral mode of communication and attend aural-oral rehabilitation programs after implantation. Children enrolled in oral communication programs demonstrate better spoken language after implantation than children enrolled in total communication programs [15]. Third, there is a long Chinese tradition of families investing in the education of their children, which is further enhanced by the one-child policy and economic growth in Mainland China. Children from small families tend to exhibit better speech perception skills and language skills [5, 15]. These differences mean that we should carefully establish appropriate predictors of speech and vocabulary development, specific to Mandarin-speaking children.

In addition, most studies have examined direct relationships, and few have examined the indirect effects of these demographic variables on speech perception and vocabulary. For example, although age at implantation is an important predictor of post-implant performance, no studies have evaluated factors that contribute to early implantation and thus, their indirect effects on speech perception and vocabulary skills. In the current study, we examined how variables appropriate in the context of Mainland China interacted with each other and therefore, which were the combined direct and indirect effects of these variables on speech perception and vocabulary development using structural equation modeling (SEM).

## Methods

### The *a priori* model

**Fig 1:**
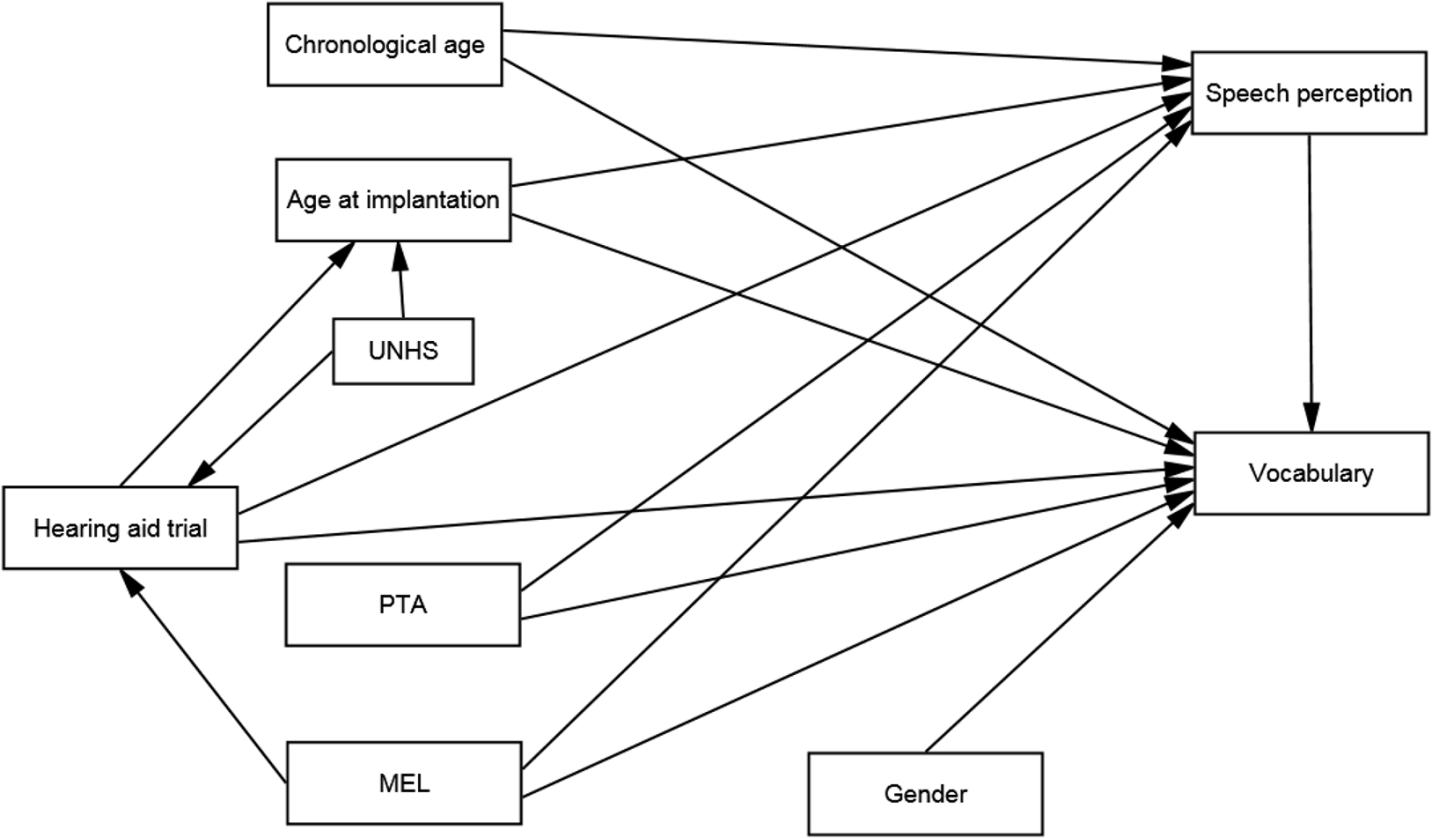
The *a priori* model tested in the current study. This is a path diagram describing the hypothesized effects of demographical factors on speech perception and vocabulary skills. Unidirectional straight arrows indicate the predicted direction of the hypothesized effect. Note: UNHS: universal newborn hearing screening, PTA: pre-implant hearing level, MEL: maternal education level

SEM can be viewed as an extension of multiple regression analysis, but it allows equation residuals to be correlated, and several dependent variables can be studied in a single SEM. Before performing SEM, a hypothesized model (i.e., an *a priori* model) describing the complex relationships among demographical factors, speech perception and vocabulary skills is required. Based on findings from previous studies, the *a priori* model was developed (Fig 1). The model rationales are discussed below.

### The effects of gender

It has been documented that girls with normal hearing (NH) ability develop a larger vocabulary and demonstrate more complex grammar than do boys of the same age [16, 17]. These advantages are also apparent in young girls with CIs [15]. However, gender effects on speech perception have not been found [5]. Therefore, it was hypothesized that gender would have a direct effect on vocabulary skills but not on speech perception outcomes.

### The effects of early age at implantation and universal hearing screening

Numerous studies on children with pre-lingual HI suggested that early implantees exhibit better speech perception [1] and expressive and receptive vocabulary skills [2]. Children implanted before the age of 2 to 3 years are more likely to develop speech perception and language abilities at a similar rate after implantation to that of age-matched children with normal hearing [18], despite a measurable gap between language age and chronological age. The influence of age is related to the presence of a “sensitive period” in the maturation of the auditory system [19]. Sharma and colleagues found that children implanted before 3.5 years of age demonstrated age-appropriate cortical auditory evoked latency responses within 6 months of CI activation, and they concluded that during the sensitive period the human central auditory system remains maximally plastic [20]. Children implanted before this age are expected to demonstrate a similar rate of growth in language skills to that of their NH peers.

Between 92% to 95% of infants born in in the United States were screened [21], leading to an increase in early diagnosis and intervention. As of 2012, universal newborn hearing screening (UNHS) was conducted in 20 of the 32 provinces in Mainland China, but access to UNHS was still constrained by a massive shortage of hearing care professionals [9]. As a result, it is not uncommon for HI to be identified after the first year of life, and intervention is not provided until children are about 2 to 3 years of age [11]. Based on these findings, it was posited that children who were screened were more likely to be implanted at an early age and early implantation would exert a direct effect on speech perception ability and vocabulary skills.

### The effects of a hearing aid trial

Since the introduction of multichannel cochlear implants in 1990, candidacy has been extended to younger children due to the advancement of implant technology and safer surgery [22, 23]. However, the candidacy requirement of a three-to-six month pre-implantation hearing aid trial (HAT) has remained unchanged. A trial that shows a potential for developing auditory, speech and language skills with hearing aids (better than or equal to the outcomes expected from a CI) will result in a recommendation to monitor the oral language development continually, instead of implantation [24]. Other benefits afforded by a trial of hearing aids and habilitation may include exposing a child to auditory cues at an early age, familiarizing a child with amplification devices and habilitation procedures, and helping parents to establish appropriate expectations about CIs [10]. Therefore, children who have undergone a hearing aid trial may exhibit better auditory skills after implantation than those who have not [10]. Unfortunately, many pediatric candidates in Mainland China did not undergo a HAT before implantation. Many children with severe to profound HI are not diagnosed until after the first year of life. Parents may opt for cochlear implantation without a HAT in order to take advantage of early implantation. Additional factors that would reduce the likelihood of a hearing aid trial included poor finances, distance from the habilitation center, failure to understand the importance of a HAT and lack of appropriate instructions from hearing healthcare providers [10]. Therefore, it was expected that having undergone UNHS would increase the chance of a child having a HAT, and that a HAT would be positively related to outcomes in speech perception and vocabulary.

### The effects of better preoperative hearing level

Several studies have reported that better preoperative hearing levels are related to better speech perception after implantation [1, 3]. However, some studies failed to find such a relationship [5, 25, 26]. Furthermore, complex relationships between CI outcomes and demographic factors, such as age at implantation, maternal educational level and duration of CI use, can impact the relationship between preoperative factors and speech performance after implantation. In the present study, the effects of pre-implant hearing level on speech perception and vocabulary were examined after controlling for other demographic factors. It was hypothesized that better pre-implant hearing would also contribute to better speech perception and vocabulary outcomes in Mandarin-speaking children with more than one year of CI use.

### The impact of higher maternal education level

Higher maternal education level (MEL) has been found to be related to better speech perception and language skills in English-speaking children with CIs [6], as well as their Mandarin-speaking peers [11]. Therefore, it was expected MEL to have a direct effect on speech perception and vocabulary skills. Furthermore, because of a lack of audiological and rehabilitative service in mainland China [9], it is also postulated that mothers with higher education level were more likely to seek help from appropriate sources so that HI could be identified early and HAT prior to implantation was therefore more likely.

### The effects of better speech perception ability

The ability to perceive sounds is the foundation for the development of language [27]. Speech perception, presumably a measure of implant benefit, is highly correlated with vocabulary and language outcomes (*r* = 0.7–0.8) [28–31]. Therefore, it was also posited that demographic factors influencing speech perception would exert an indirect effect on vocabulary competency via their effects on speech perception.

The relationships discussed above could be summarized as follows (Fig 1). First, MEL, age at implantation, whether a child had undergone a hearing aid trial (HAT), and pre-implant hearing level were expected to exert direct effects as well as indirect effects on vocabulary via their impacts on speech perception. Second, it was expected that female participants would exhibit better vocabulary skills, but that gender would not make a difference in speech perception. Third, when mothers had higher educational level, and when the HI was identified early via UNHS, the age at which children received implantation would be lower. Finally, higher maternal education level and having attended UNHS would increase the chance of having a HAT prior to CI.

Besides the above facts, chronological age was also included in the *a priori* model as a covariate of speech perception and vocabulary outcomes [32, 33]. Other factors that were expected to affect outcomes were included as subject selection criteria. These included the exclusion of subjects with comorbid disability, with low cognitive ability, exposed to other spoken dialects and who do not use an oral only mode of communication [7, 34–36].

### Participants

A convenience sample of 115 children with CIs was recruited at the General Hospital of People’s Liberation Army from 2012 to 2014. Descriptive statistics are presented in Table 1. All children had been implanted before the age of 5 and had used CIs for about 1 to 3 years. The children also met the following inclusion criteria: (1) they were fitted with unilateral CIs, which is the current norm in Mainland China; (2) they exhibited normal cognitive abilities, which were screened using the Griffiths Mental Development Scales for children below 3 years of age or the Hiskey-Nebraska Test of Learning Aptitude for children above 3 years of age [37]; (3) they exhibited normal inner ear structure, and no auditory nerve absence or deficiency, as reported in radiological findings; (4) they exhibited no oral-motor disorders, behavioral disorders or neurodevelopment disorders such as autism; (5) they used the oral mode of communication; (6) they spoke Mandarin as the primary language in the family and were exposed to Mandarin no less than 90% of the time in daily situations; and (7) they had all received no less than one year of rehabilitation.

**Table 1:** Participant characteristics (N = 115).

	Mean	Range	SD
Pure-tone average thresholds in the better ear (0.5, 1, 2, 4 kHz)(dB)	97.80	80.25–112.75	10.36
Chronological age (years)	4.16	2.50–7.09	1.05
Age at implantation (years)	2.67	0.69–5.00	1.08
Duration of CI use (years)	1.42	0.83–3.23	0.73
Maternal education level (years)	10.34	0–19	3.56

Among the participants in the present study, 22% had undergone UNHS, and 60.4% had tried HA before implantation. Only five children had been using a hearing aid (HA) in the non-implanted ear since implantation, and they were tested with both CIs and HA on. This study was approved by The Human Research Ethics Committee for Non-Clinical Faculties, the University of Hong Kong. All participants voluntarily joined this study with written informed consents obtained from their parents.

## Materials

Speech perception and vocabulary skills were measured using two batteries of speech perception and vocabulary tests. The battery of speech perception tests include the Mandarin Meaningful Auditory Integration Scale (MAIS) [38], the Mandarin Early Speech Perception test (MESP) [39], and the Mandarin Pediatric Speech Intelligibility test (MPSI) [40]. These tests have been standardized for normal-hearing children aged between 0 to 72 months in mainland China.

The Mandarin MAIS is a 10-item parent interview tool used to assess spontaneous listening behaviors in an everyday listening environment. The 10 items mainly tap three different aspects of listening skills: 1) device bonding (questions 1–2), which assesses the child’s vocalization changes with device use; 2) sound detection (questions 3–6), which evaluates the child’s spontaneous responses to sounds; and 3) perception (questions 7–10), which refers to the ability to derive meaning from sounds.

The MESP is a closed-set, norm-referenced, and software-administered speech perception test. It is composed of six categories: Speech detection (Category 1), pattern perception (Category 2), spondee perception (Category 3), vowel perception (Category 4), consonant perception (Category 5), and tone perception (Category 6). The MESP is a hierarchical test, with category 1 being the easiest and Category 6 being the hardest. The child proceeds to the next category if the score obtained using the current category is significantly higher than chance, as defined by Zheng and colleagues.

The MPSI is a closed-set, norm-referenced, and software-administered sentence perception test. The MPSI is administrated in quiet or in the presence of a competition sentence at signal-to-noise ratios (SNRs) of +10, +5, 0, -5, and-10 dB SNR. The noise is a competing sentence that is randomly chosen by the software from a pool of 12 competing sentences. There are two sets of test sentences in the MPSI. Each set includes three pairs of sentences. Each pair has the same subject with different verbs and objects. The target sentence was presented at 65 dB (A) via a loudspeaker located in front of the child (0° azimuth) at a distance of one meter. A competing sentence was delivered via a loudspeaker behind the participants at 180° azimuth at a distance of one meter. Testing starts with the quiet condition and then progressed from + 10 dB SNR to-10 dB SNR in 5-dB steps. When a correct score significantly higher than chance (i.e., 41.7% at *p* < 0.05) was obtained, the next test condition was administered [40].

The battery of vocabulary tests included the Chinese Communicative Developmental Inventory–Putonghua version-short form (CCDI) [16] and the Mandarin Expressive and Receptive Vocabulary Test (MERVT) [33]. The CCDI [16], which originated from the MacArthur-Bates Communicative Development Inventory (MCDI), is a parent report measure for the appraisal of vocabulary skills in Mandarin-speaking infants or toddlers aged 8 to 30 months. The psychometric characteristics of this form have been evaluated and the norming study was conducted in Beijing [16].

The CCDI consists of two components: Words and Gestures and Words and Sentences. The Words and Gestures component is a vocabulary checklist of 106 words. During a face-to-face parent interview, items that a child can say and/or understand can be checked, yielding an expressive vocabulary score and a receptive vocabulary score (i.e., CCDI-G-E and CCDI-G-R). The Words and Sentences component is a 92-word checklist. Caregivers are asked to determine the words that the child can say, yielding an overall vocabulary production score (i.e., CCDI-S-E).

The MERVT test is designed for direct assessment of expressive and receptive vocabulary skills. The test was normed on 245 normal-hearing children aged from 1.5 to 4 years in Beijing, China. The psychometric characteristics have been evaluated [33]. The MERVT consists of two scales: the receptive vocabulary scale and the expressive vocabulary scale. The receptive vocabulary scale is a closed-set task and composed of 88 words. Participants are asked to choose the target among four illustrations that are depicted on a picture plate. The other 3 illustrations serve as semantic distracters. The expressive vocabulary scale consists of 73 items, and participants are asked to name them. The order of words on MERVT is arranged according to their level of difficulty. The test stops when wrong responses to 5 consecutive items are given [33].

## Procedures

Consent and demographic information were obtained first, followed by the collection of demographic information. The Mandarin MAIS and the CCDI were conducted in a face-to-face interview with a caregiver. The MESP, the MPSI, and the MEVRT were administered to each participant in a test room with noise level below 40dB (A). All the measures were conducted in a random order. Time required to complete these tests varied as tests administered to children are hierarchical in nature (i.e., the MESP, the MPSI and the MERVT). The maximum time spent was 2.0 hours with most participants being tested for less than 1.5 hours. Frequent breaks were offered throughout the session. Most of the children finished testing within one day.

## Results

Due to the relatively small sample size (N = 115) in comparison to the number of measures used, the speech perception tests and vocabulary tests were first reduced to a single speech perception score and a single vocabulary score, respectively, using principal components analysis (PCA). The results were then used to test the hypotheses proposed in the *a priori* model.

### Principal component analysis

Principal component analysis (PCA) were conducted on four speech perception scores and five vocabulary scores. First, the Kaiser-Meyer-Olkin (KMO) measure verified the sampling adequacy for the analyses. The KMO statistic ranges from 0 to 1. A value below 0.5 indicates that the sample size is too small for performing PCA [41]. In the current study, KMO values of 0.74 for speech perception measures and 0.82 for vocabulary measures, exceeded the criterion value of 0.5, indicating that the sample size was adequate for PCA.

Next, if all the tests measure the same underlying dimension, tests would correlate with each other. A significant value of Bartlett’s test suggests that all correlations between tests overall are significantly different from an identity matrix where the tests did not correlated at all. In the current study, Bartlett’s test for speech perception measures χ^2^(6) = 346.97, *p*<0.001 and for vocabulary measures χ^2^(10) = 504.45, *p*<0.001, indicated that correlations between tests were sufficiently large for PCA.

Third, PCA generates as many components as test variables, but not all components are important. Components that have an eigenvalue greater than the Kaiser’s criterion of 1 were retained [42]. In the current study, only the first component met this criterion and explained most of the variance (76%) of speech perception scores. Similarly, only one component for vocabulary scores met this criterion and explained 78% of the variance of vocabulary scores. Table 2 shows the factor loadings of the first component for the speech perception and vocabulary respectively using PCA. The factor loading ranges from 0 to 1 and it could be thought of as the Pearson correlation between a latent factor (i.e., the speech perception skill) and a variable (i.e., each test) [41].

**Table 2:** Summary of PCA results for speech perception and vocabulary tests (N = 115).

Speech perception			Vocabulary	
Test	Factor loadings	Test	Factor loadings
MAIS	0.78	MERVT-E	0.88	
MESP	0.94	MERVT-R	0.87	
MPSI-Q	0.93	CCDI-G-R	0.84	
MPSI-N	0.81	CCDI-G-E	0.89	
		CCDI-S-E	0.92	

Note: MAIS: the Mandarin Meaningful Auditory Integration Scale. MESP: the Mandarin Early Speech Perception test. MPSI-Q: the Mandarin Pediatric Speech Intelligibility in quiet test. MPSI-N: the Mandarin Pediatric Speech Intelligibility in noise test. MERVT-E: the Mandarin Expressive and Receptive Vocabulary Test—Expressive scale. MERVT-R: the Mandarin Expressive and Receptive Vocabulary Test—Receptive scale. CCDI-G-R: the Chinese Communicative Developmental Inventory-Words and Gestures-Receptive scale. CCDI-G-E: the Chinese Communicative Developmental Inventory-Words and Gestures-Expressive scale CCDI-S-E: the Chinese Communicative Developmental Inventory-Words and Sentences-Expressive scale.

Then, the following equations were used to generate a single composite speech perception score and a single composite vocabulary score for each child [41].

Speech perception: Yi = 0.78x_1i_+0.94x_2i_+0.93x_3i_+0.81x_4i_

Vocabulary: Zi = 0.88v_1i_+0.87v_2i_+0.84v_3i_+0.89v_4i_+0.92v_4i_

Where a single composite speech perception score and a single composite vocabulary score for the participants *i* are donated by Yi and Zi respectively. Percent correct scores for the MAIS, the MESP, the MPSI-Q, the MPSI-N, the MERVT-E, the MERVT-R, the CCDI-G-R, the CCDI-G-E, and the CCDI-S-E for participant *i* are donated by the x_1i_, x_2i_, x_3i_, x_4i_, _V1i, V2i, V3i, V4i, V5i_ respectively. Coefficients of xi and vi are the principal component loading (Table 2.) for each speech perception or vocabulary test.

### Modeling of speech perception and vocabulary outcomes

**Fig 2:**
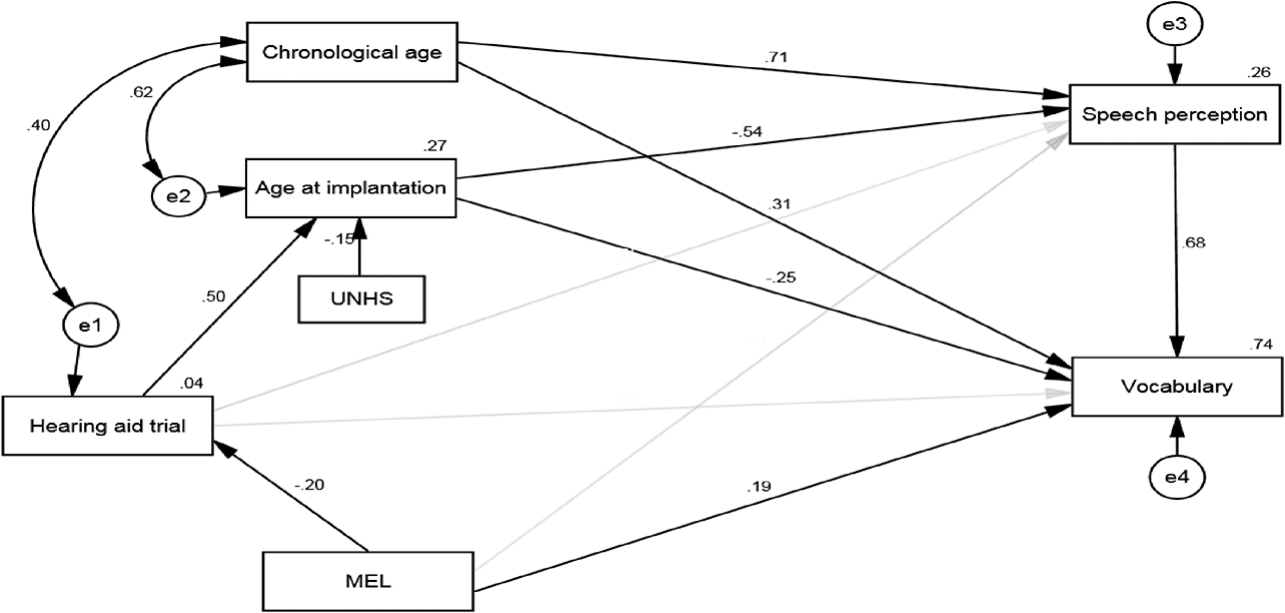
Final model describing the relationships among demographical variables, speech perception and vocabulary ability. Standardized path coefficients are presented at the midpoint of the unidirectional arrow paths (N = 115). Values noted besides the endogenous variables represent the percentage of variance accounted for each variable. The insignificant paths are shown in gray and their path coefficients are not presented. *e* represents the residuals in the prediction of endogenous factors from exogenous factors. Note: UNHS: universal newborn hearing screening, PTA: pre-implant hearing level, MEL: maternal education level.

Before SEM was conducted, the correlation matrix of the variables was used to explore the possibility of simplifying the model (Table 3). Several modifications were made to the *a priori* model based on results from the correlation matrix among demographical factors, speech perception, and vocabulary outcomes. First, gender and PTA were excluded from the *a priori* model (Fig 2) as they were not significantly correlated with speech perception, vocabulary or other demographic variables. Second, the path from UNHS to HAT was also excluded from the *a priori* model as UNHS and HAT were not related (Table 3). Third, additional relationships were specified because they were not hypothesized before the correlation analysis. We correlated the error term of HAT (i.e., e^1^) and the error term of age at implantation (i.e., e^2^) with chronological age as they were significantly correlated (Table 3). Every endogenous (dependent) variable is expected to be associated with an error term, which represents the residuals in the prediction of endogenous factors from exogenous (independent) factors. For example, e^2^ shown in Fig 2 represents the residuals in the prediction of age at implantation (the endogenous variable) from UNHS and HAT (the exogenous variables).

**Table 3:** Correlation coefficients among variables in the *a priori* model.

	Gender	UNHS	CA	AAI	PTA	MEL	HAT	Speech perception scores
UNHS	-0.14							
CA	-0.06	-0.19*						
AAI	-0.03	-0.27**	0.75**					
PTA	-0.04	0.07	0.06	0.08				
MEL	-0.20	-0.10	-0.14	-0.22*	-0.12			
HAT	0.12	-0.02	0.41**	0.50**	0.12	-0.22*		
Speech perception score	-0.15	-0.03	0.31**	-0.02	-0.15	0.13	0.04	
Vocabulary score	-0.14	-0.06	0.30**	-0.09	-0.16	0.30**	-0.03	0.81**

Note: UNHS: universal newborn hearing screen; CA: chronological age; AAI: age at implantation; PTA: pre-implant hearing level; MEL: maternal education level; HAT: hearing aid trial.

*p<0.05

**p<0.01.

The data were then checked to ensure that they met the four assumptions underlying maximum likelihood (ML) estimation for SEM: the distribution of the data is univariate and multivariate normal; the sample is sufficiently large; the model is based on substantive theory and missing data is completely random. First, all variables had skewness and kurtosis values of between ±3.0, ranging from-1.00 to 1.06 [43] and a Mardia’s kurtosis value of 2.20 and a critical ratio of 1.05 (*p*>0.05) [44], which meant that the data were univariate and multivariate normally distributed. Second, the sample size of this study was 115, which was acceptable for a SEM model with 21 free parameters [42]. Third, the hypothesis and causal relationships in the model were generated based on findings in the literature, as reviewed in the introductory section. The third assumption underlying model validity, therefore, was met. Lastly, the fourth assumption was also met as there were no missing data in the model.

The Analysis of Moment Structure 22 (Amos 22) software was used to perform the SEM. The final model (Fig 2) was examined for goodness-of-fit. Several measures of goodness-of-fit of the final model were selected and all these measures indicated a good model fit. First, the model yielded a chi-square value of 9.91, *p* > 0.05, which suggested that the theoretical model (i.e., the final model) did not differ significantly from the data driven model. Second, since the chi-square statistic is sensitive to sample size, incremental fit indices (i.e., TLI and CFI) were also examined. The incremental fit indices reflect the increase in model fit in relation to a null model where all variables are uncorrelated. A value greater than 0.95 is indicative of a good fit [45]. Both the TLI (0.97) and the CFI (0.99) obtained here suggested that the final model represented a good fit to the model. Lastly, the Root Mean Square Error of Approximation (RMSEA) was used to compare the covariance matrix derived from the final model, adjusting for model complexity, and the actual covariance matrix [46]. A value of 0.06 obtained here also indicated a good fit. Due to the good fit of data to the model, post-hoc modifications were not needed [47].

In addition to overall model fit, path coefficients also provide information regarding the direct and indirect effects. The direction of the arrow in Fig 2 implies the flow of the causal effect and the impact of one variable on another. Standardized path coefficients (i.e., the direct effect of a variable on the other) varied between-1 and +1 and could be interpreted in the same way as standardized multiple regression coefficients, indicating the amount of standard deviations (SD) a dependent variable will change, per standard deviation increase in the predictor variable. A value less than 0.1 indicates a “small” effect, a value between 0.1 to 0.3 suggests a “medium” effect, and a value greater than 0.3 indicates a “large” effect [48].

Table 4 shows the standardized total, direct and indirect effects of each variable on speech perception skills. These effects could also be interpreted in the same way as standardized path coefficients or standardized multiple regression coefficients (see last paragraph). For example, early age at implantation predicted better speech perception skills, with a standardized direct effect of-0.54. This means that a SD decrease in age at implantation was associated with a 0.54SD increase in the composite speech perception score. Effects of a HAT on speech perception were mediated by age at implantation, with a standardized indirect effect of-0.27. MEL had indirect effects on speech perception as well. That is, children with higher MELs were less likely to undergo a HAT and thus tended to receive CIs at an earlier age, which led to better speech perception performance. However, the indirect effect of MEL on speech perception was negligible (standardized indirect effect of 0.04). Similarly, UHNS exercised an indirect effect on speech perception skills through its effects on age at implantation, with a very small standardized indirect effect of 0.08. The final model accounted for 26% of the variance in speech perception scores.

**Table 4:** Standardized direct, indirect, and total effects of variables on speech perception (N = 115).

Variable	Direct effect	Indirect effect	Total effect
Age at implantation	-0.54**	-	-0.54**
HAT	0.05	-0.27**	-0.22*
MEL	0.11	0.04*	0.16*
UNHS	-	0.08*	0.08*

* *p* <0.05

** *p* <0.01

Note: HAT: hearing aid trial; MEL: maternal education level; UNHS: universal newborn hearing screening.

Table 5 shows the standardized total, direct and indirect effects of each variable on vocabulary skills. Age at implantation affected vocabulary directly and indirectly via speech perception, with a total standardized effect of 0.62. MEL had both direct and indirect effects on vocabulary, with a total standardized effect of 0.32. The magnitude of standardized direct and indirect effects of MEL on vocabulary was similar, which was around 0.15. Although HAT did not directly influence vocabulary scores, it affected vocabulary scores through its effects on age at implantation (total standardized indirect effect = 0.27). UNHS exercised an indirect effect on vocabulary scores through its effects on age at implantation although this effect was negligible (standardized indirect effect = 0.09). Among all the variables, speech perception had the largest effects on vocabulary, with a standardized direct effect of 0.68. The final model accounted for 74% of the variance in vocabulary scores.

**Table 5:** Standardized direct, indirect, and total effects of variables on vocabulary (N = 115).

Variable	Direct effect	Indirect effect	Total effect
Age at implantation	-0.25**	-0.37**	-0.62**
HAT	-0.01	-0.27**	-0.29*
MEL	0.19*	0.13*	0.32*
UNHS	-	0.09*	0.09*
Speech perception	0.68**	-	0.68**

* *p* <0.05

** *p* <0.01

Note: HAT: hearing aid trial; MEL: maternal education level; UNHS: universal newborn hearing screening.

However, several paths in the *a priori* model were found to be insignificant in the final model (i.e., *p*>0.05). These paths are highlighted in gray in Fig 2, including the path from HAT to speech perception, the path from HAT to vocabulary scores, and the path from MEL to speech perception. In other words, whether a child had undergone a HAT did not significantly affect speech perception and vocabulary scores and high MEL did not significantly contribute to speech perception skills.

## Discussion

This study examined the contribution of various factors to variations in speech perception and vocabulary abilities in Mandarin-speaking children with CIs. These factors were considered simultaneously in the *a priori* model, thus allowing hypothesized relationships to be tested. A total of 74% of the variance in vocabulary scores and 26% of the variance in speech perception scores was explained by variables in the final model. The findings demonstrated that early implantation significantly contributed to speech perception directly, while HAT, MEL, and UNHS had only indirect effects on speech perception. In addition, both age at implantation and MEL had direct and indirect effects on vocabulary skills while UNHS and HAT had indirect effects on vocabulary skills. These relationships are elaborated below.

### Age at implantation

The age at which children had received CIs significantly affected their speech perception and vocabulary outcomes. That is, children who had received their CIs early achieved higher speech perception and vocabulary scores, and these effects were large. The predictive power of age at implantation with respect to speech perception and vocabulary has also been demonstrated by other investigators [49–51]. Besides direct effects, age at implantation could also indirectly affect vocabulary skills via speech perception. In other words, speech perception mediated the effects of age at implantation on vocabulary, in addition to the direct effects. These findings suggested that early implantation provided access to spoken Mandarin during the sensitive period for auditory learning, which in turn affected language development.

Factors contributing to early implantation were also examined in the current study. In the *a priori* model, it was hypothesized that high MEL and UNHS would contribute to early implantation. However, results revealed that MEL did not significantly affect age at implantation. Although UNHS contributed to early implantation, the effect was small, with a standardized effect of-0.15. Although previous studies have shown that early identification leads to early implantation, the situation in mainland China is such that intervention may not be provided immediately following identification. Moreover, hearing services may be prohibitive for children living in rural areas and travelling to a metropolis to obtain services may be expensive and time-consuming. Thus, the impact of UNHS on age at implantation is minimal in mainland China.

### Hearing aid trial

The use of hearing aids prior to implantation and experience in an aurally based therapy program before implantation may be beneficial to speech perception development [52]. Chen and colleagues showed that children who had undergone a HAT exhibited better auditory skills during the first year of CI use than those who had not [10]. However, in the present study, we did not find any relationship between having undergone a HAT and composite speech perception and vocabulary scores. Chen and colleagues reported that although children who had undergone a HAT performed significantly better in sentence perception in noise scores compared to those who had not undergone a HAT, this factor did not significantly affect tone perception [11]. Therefore, it was reasonable to speculate that having undergone a HAT might also have an impact on individual tests (i.e., sentence perception in noise) in children, instead of the overall speech perception or vocabulary scores. This hypothesis should be verified by future research.

Although a HAT may not significantly affect overall speech perception and vocabulary skills, it is still critical in determining implant candidacy. In the *a priori* model, it was hypothesized that higher MEL and UNHS would increase the chances of having undergone a HAT. Results showed that UNHS did not significantly determine whether the children had undergone a HAT. This again could be attributed to limited audiological services available in Mainland China, such that early intervention might not have been possible for the children who underwent UNHS. In addition, contrary to our hypothesis, it was found that children from families with lower MEL were more likely to have undergone a HAT. As mothers with lower MEL were more likely to be involved in a CI donation program that required candidates to have a HAT, this finding is not surprising. In spite of being recommended to undergo a HAT before implantation, children who receive their CIs at their own expense are less likely to have a HAT before implantation than children in donation programs, as discussed in the Introduction. It was unfortunate that even though the mean interval between the time at identification and the time at implantation was sufficiently long to allow a HAT (mean = 1.63 years), not all children had undergone a HAT to attain consistent auditory input prior to implantation.

### Maternal educational level

It was hypothesized that high MEL would contribute to speech perception and vocabulary skills. Results revealed that MEL only affected vocabulary skills after implantation and did not significantly increase the chances of a HAT before implantation or improving speech perception after implantation. The significant relationship between vocabulary and MEL could be attributed to two factors: first, mothers with higher MEL tend to be less directive, talk more, and use a more varied vocabulary in conversing with their children than do mothers with lower MEL [53]; and second, mothers with higher educational levels are more likely to seek help from appropriate sources, which is especially important in a country with a lack of audiological and rehabilitation services. Parents of implantees living in Western societies have usually received more years of education than their Mandarin-speaking peers. As a result, studies conducted on these children either did not include MEL as a variable or found it not significantly related to speech perception or language outcomes [12]. However, considering the limited audiological resources and the large range of MELs, this factor may play a more important role in language development in developing countries than in societies where hearing health care is well developed.

### Gender and pre-implant hearing level

Although gender effects on vocabulary development have been reported in young children with NH, these differences are usually small in magnitude and disappear around 5 years of age [17, 54]. In the present study, we did not find gender effects on outcomes with CIs. The fact that SEM considers complex causal relationships and errors associated with measurements means that factors that are erratic in their relationships with these variables have probably been ruled out.

Better pre-implant hearing level also did not contribute to better speech perception and vocabulary in the current study. Although Mondain and Colleagues reported that children with more residual hearing attained better speech perception skills after implantation [3], the children in their studies had used CIs for less than a year. As experience in the use of CIs increases, the influence of preoperative hearing level on speech perception and language may diminish [22]. In addition, as a large number of children in the present study had not undergone a hearing aid trial, the use of residual hearing was probably too minimal to have an impact. This might also have contributed to the insignificant relationship between the PTA and speech perception.

It is notable that only the effects of demographic factors on early speech perception and vocabulary development in Mandarin-speaking children with CIs were investigated in this study. Although these demographic factors accounted for a significant proportion of the variance (i.e., 20% to 30%) in early speech perception and vocabulary skills, they did not account for all the variations in outcomes. Device and surgical factors, such as insertion depth, electrode-neuron interface, dynamic range, neural survival, signal processing might also affect speech perception and vocabulary development in children with CIs [5]. In addition, Pisoni and Cleary [55, 56] found that pediatric implantees demonstrated slower verbal rehearsal speeds and shorter working memory spans than their peers with NH [55, 56]. They also reported that pediatric implantees with faster verbal rehearsal and shorter working memory spans had better speech perception skills [5]. Therefore, future studies, may investigate the effects of individual differences in devices, surgeries, and specific cognitive (e.g., learning, memory, and attention) on the variability in CI benefits.

## Implications

Geers and colleagues studied language development in 181 English-speaking children with 4 to 7 years of CI use. They found that higher nonverbal intelligence, smaller family size, higher socio-economic status, being female, oral mode of communication, and better speech perception skills after implantation were related to better spoken language outcomes but they did not find outcomes related to age at implantation [5]. However, in the present study, lower age at implantation, higher MEL, and better speech perception after implantation were direct predictors of better vocabulary development in Mandarin-speaking children after implantation whereas gender, and HAT were not. In addition to accounting for the cognitive ability of participants, almost all the families used the oral mode of communication and had a simple structure as a result of the “one-child” policy in mainland China. Although these differences made comparison between findings from English-speaking and Mandarin-speaking children difficult, they highlighted the importance of localizing the research design specifically to the context of mainland China. In other words, studies of CI outcomes should consider accounting for environmentally appropriate factors.

Furthermore, findings from the current study are expected to inform stakeholders, policy makers, parents and clinicians on how to maximize CI outcomes. First, even when the effects of speech perception skills were accounted for, high MEL significantly contributed to vocabulary scores. Therefore, additional support should be provided to children with low MEL. Second, gender and PTA did not have any direct or indirect effects on speech perception and vocabulary. These factors therefore should not be criteria for considering candidacy as many donation programs in Mainland China presently do. Lastly, in the context of sparse availability or commitment to follow-up audiological care, UNHS does not guarantee a HAT before implantation or early implantation in Mainland China. Given that the average time interval between identification and implantation was usually more than 1.5 years in the present study, policy makers and parents should consider implementing good quality early intervention to guarantee the best outcomes.
